# Combined B, T and NK Cell Deficiency Accelerates Atherosclerosis in BALB/c Mice

**DOI:** 10.1371/journal.pone.0157311

**Published:** 2016-08-26

**Authors:** Fei Cheng, Laura Twardowski, Kurt Reifenberg, Kerstin Winter, Antje Canisius, Eva Pross, Jianglin Fan, Edgar Schmitt, Leonard D. Shultz, Karl J. Lackner, Michael Torzewski

**Affiliations:** 1 Dr. Margarete Fischer-Institute of Clinical Pharmacology, Stuttgart and University of Tübingen, Tübingen, Germany; 2 Department of Laboratory Medicine, Robert-Bosch-Hospital, Stuttgart, Germany; 3 Center for Preclinical Research, German Cancer Research Center, Heidelberg, Germany; 4 Institute of Clinical Chemistry and Laboratory Medicine, University Medical Center, Johannes Gutenberg University Mainz, Mainz, Germany; 5 Department of Molecular Pathology, Interdisciplinary Graduate School of Medicine and Engineering, University of Yamanashi, Yamanashi, Japan; 6 Institute for Immunology, University Medical Center, Johannes Gutenberg University Mainz, Mainz, Germany; 7 The Jackson Laboratory, Bar Harbor, Maine, United State of America; Tulane University, UNITED STATES

## Abstract

This study focused on the unique properties of both the *Ldlr* knockout defect (closely mimicking the human situation) and the BALB/c (C) inbred mouse strain (Th-2 slanted immune response). We generated two immunodeficient strains with severe combined B- and T-cell immunodeficiency with or without a complete lack of natural killer cells to revisit the role of adaptive immune responses on atherogenesis. C-*Ldlr*^*-/-*^ *Rag1*^*-/-*^ mice, which show severe combined B- and T-cell immunodeficiency and C-*Ldlr*^*-/-*^ *Rag1*^*-/-*^ *Il2rg*^*-/-*^ mice, which combine the T- and B-cell defect with a complete lack of natural killer cells and inactivation of multiple cytokine signalling pathways were fed an atherogenic Western type diet (WTD). Both B6-*Ldlr*^*-/-*^ and C-*Ldlr*^*-/-*^ immunocompetent mice were used as controls. Body weights and serum cholesterol levels of both immunodeficient strains were significantly increased compared to C-*Ldlr*^*-/-*^ controls, except for cholesterol levels of C-*Ldlr*^*-/-*^ *Rag1*^*-/-*^ double mutants after 12 weeks on the WTD. Quantification of the aortic sinus plaque area revealed that both strains of immunodeficient mice developed significantly more atherosclerosis compared to C-*Ldlr*^*-/-*^ controls after 24 weeks on the WTD. Increased atherosclerotic lesion development in C-*Ldlr*^*-/-*^ *Rag1*^*-/-*^ *Il2rg*^*-/-*^ triple mutants was associated with significantly increased numbers of macrophages and significantly decreased numbers of smooth muscle cells compared to both C-*Ldlr*^*-/-*^ wild type and C-*Ldlr*^*-/-*^ *Rag1*^*-/-*^ double mutants pointing to a plaque destabilizing effect of NK cell loss. Collectively, the present study reveals a previously unappreciated complexity with regard to the impact of lymphocytes on lipoprotein metabolism and the role of lymphocyte subsets in plaque composition.

## Introduction

Manipulation of the immune system by a variety of means has been used to unravel the roles of cellular and humoral immunity in the pathogenesis of atherosclerosis [1]. Although the absence of autoantibodies and T lymphocytes did not influence [2] or only played a minor role [3] in the extent of aortic atherosclerotic lesions in Apoe-deficient mice, studies in Ldlr deficient mice suggest that T lymphocytes exert pro-inflammatory effects early in atherogenesis [4]. In contrast, subsets of B lymphocytes and/or antibodies are protective against atherosclerosis both in Apoe-[5] and Ldlr-[6] deficient mice.

Unlike conventional T cells that recognize peptide antigens presented by the classical MHC class I or class II molecules, natural killer T (NKT) cells are specific for glycolipid antigens presented by the MHC class I-like molecule CD1 [7]. CD1d-restricted NKT cells are pro-atherogenic in both Apoe- [8] and Ldlr- [8–10] deficient mice.

Depending on the local milieu of cytokines, CD4^+^ T lymphocytes differentiate into a T-helper (Th) 1 or Th 2 lineage. Cytokines associated with a pro-inflammatory Th-1 response (interferon (IFN)-γ and interleukin (IL)-12) play a role in atherogenesis while Th2 associated cytokines including IL-10 are protective against lesion development [11]. It is also known that severe hypercholesterolemia induces a switch from Th1 to Th2 development in Apoe-deficient mice resulting not only in the formation of IgG1 autoantibodies to oxidized LDL, but also in the appearance of Th2-type cytokines in the atherosclerotic lesions [12]. The impact of a Th1 versus Th2 immune response on atherogenesis was further detailed in the context of Apoe-deficient mouse strains, which either had a C57BL/6 (B6) background, producing predominantly Th1 helper cells, or which had a BALB/c (C) background, leaning toward Th2 dominated immune responses [13]. However, as Apoe-deficient mice exhibit an impaired immune response during infection with *Listeria monocytogenes* [14] general concerns over using this model to evaluate immune response in atherosclerosis have been raised, and it has been demonstrated that Ldlr-deficient mice did not exhibit any potentially confounding perturbations in immune response [4]. In addition, since atherosclerotic lesions in Ldlr-deficient mice do not develop spontaneously as in Apoe-deficient animals but are inducible under Western type diet (WTD), and since the serum lipoprotein profile of Ldlr-deficient animals is characterized by high-level LDL rather than by chylomicrons and VLDL (as in Apoe-deficient mice) [15–17], Ldlr-deficient mice represent a more physiological model of atherogenesis compared to Apoe-deficient mice.

In the present study, we studied the unique properties of both Ldlr-deficiency (closely mimicking the human situation) and the BALB/C mouse strain (Th2 slanted immune response) and generated two immunodeficient strains with severe combined B- and T-cell immunodeficiency with or without a complete lack of natural killer cells to revisit the role of adaptive immune responses on atherogenesis.

## Materials and Methods

### Mouse Strains and Dietary Induction of Atherosclerosis

The B6-Ldlr^-/-^ (official designation: B6.129S7-*Ldlr*^*tm1Her*^/J, Stock No 2207) knockout strain was purchased from The Jackson Laboratory (TJL, Bar Harbor, Maine, USA).

Generation of the BALB/c-Ldlr^tm1Her^ (internal designation C-Ldlr^-/-^) mice has been described in Spencer et al[18] and homozygous C.129S7-*Ldlr*^*tm1Her*^ (C.Ldlr^-/-^) breeder mice were obtained from the animal care facility of the University of Washington. C-Ldlr^-/-^ mice carry the atherosclerotic *Ldlr*^*tm1Her*^ allele on the inbred BALB/c background which is prone to Th2 immune responses.

C.129S7-*Ldlr*^*tm1Her*^ *Rag1*^*tm1Mom*^ (internal designation C-Ldlr^-/-^ Rag1^-/-^) double mutants were generated by crossing the C-Ldlr^-/-^ strain to C-Rag1^-/-^ mice [19] (generously provided by the Department of Immunology of the University Medical Center, Mainz) and intercrossing the resulting C-Ldlr^+/-^ Rag1^+/-^ F1 mice. C-Ldlr^-/-^ Rag1^-/-^ mice combine atherogenesis susceptibility with a severe combined B- and T-cell immunodeficiency.

C.129S7-*Ldlr*^*tm1Her*^ *Rag1*^*tm1Mom*^ *Il2rg*^*tm1Wjl*^ (internal designation C-Ldlr^-/-^ Rag1^-/-^ Il2rg^-/-^) triple knockout mutants were generated by crossing C-Ldlr^-/-^ mice to BALB/c.Cg-*Rag1*^*tm1Mom*^ *Il2rg*^*tm1Wjl*^ mice (internal designation C-Rag1^-/-^ Il2rg^-/-^), raised by L.D.S at The Jackson Laboratory, crossing the resulting C-Ldlr^+/-^ Rag1^+/-^ Il2rg^+/-^ hybrids to the C-Rag1^-/-^ Il2rg^-/-^ parental strain until homozygosity was reached at the Rag1 and Il2rg loci and intercrossing the resulting C-Ldlr^+/-^ Rag1^-/-^ Il2rg^-/-^ mice. C-Ldlr^-/-^ Rag1^-/-^ Il2rg^-/-^ mice are prone to atherosclerosis, are deficient in mature T-cells, B-cells and natural killer cells and have inactivation of the IL-2, IL-4, IL-7, IL-9, IL-15, and IL-21 cytokine signalling pathways [20].

Genetic authenticity of all strains was monitored commercially (KBioscience, Hoddesdon, UK) using the SNP-based marker set previously developed by The Jackson Laboratory [21].

All mice were maintained at the Central Laboratory Animal Facility of the University Medical Center of Mainz, Germany, under specific pathogen free conditions in accordance with standard animal care requirements and maintained on a 12/12 hour light-dark cycle. Water and food were given *ad libitum*. Female C-Ldlr^-/-^ Rag1^-/-^ and C-Ldlr^-/-^ Rag1^-/-^ Il2rg^-/-^ knockout mice, as well as female C-Ldlr^-/-^ and B6-Ldlr^-/-^ mice were placed on a pro-atherogenic Western-type diet (WTD, Ssniff Spezialdiäten GmbH, Soest, Germany) at an age of eight weeks. The WTD contained 21% (wt/wt) fat and 0.15% (wt/wt) cholesterol and was administered for a time span of either 12 or 24 weeks, respectively.

All animal work performed in this study was conducted according to the national guidelines and was reviewed and confirmed by an institutional review board/ethics committee headed by the local animal welfare officer (Prof. Kempski) of the University Medical Center Mainz, Germany. The animal experiments were finally approved by the responsible national authority, which is the National Investigation Office Rheinland-Pfalz (Koblenz, Germany). The Approval ID assigned by this authority is AZ 23 177-07/G 07-1-003.

### Lipoprotein and Analysis of Murine Sera

Murine sera were diluted 1:3 prior to quantitative cholesterol and triglyceride analyses. Quantitative cholesterol determinations were conducted using a colorimetric assay (CHOP-PAP, Roche^®^ Diagnostics, Mannheim, Germany). Triglycerides were determined by quantifying free glycerine originating from hydrolytic cleavage (GPO-PAP, Roche Diagnostics). Additionally, lipoproteins were isolated by small-volume sequential ultracentrifugation with a Beckman TLA100.2 rotor as described. Isolated individual density fractions and whole sera were resolved by electrophoresis in 1% agarose gels. The gels were dried and stained with Fat Red 7B to identify lipoproteins containing neutral lipid [22]. Lastly, plasma lipoprotein profiles were analyzed by fast protein liquid chromatography (FPLC) gel filtration as described previously [23].

### Tissue Preparation and Quantification of Atherosclerotic Lesions

Mice fed with WTD were sacrificed by exposure to carbon dioxide and peritoneal cavities were opened. The lung, liver, small intestine, kidney, spleen, heart and aorta were resected en bloc and fixed in 4% PBS-buffered formaldehyde. Hearts were sequentially cut into a total of 30 (3 μm thick) sections around the aortic sinus. Out of 30 sections, every fifth slide was stained with trichrome and computer-assisted measurement of plaque size was performed as described previously [24]. The remaining aortic sinus and spleen sections were used for further immunohistochemistry and histochemistry.

### Immunohistochemistry and Histochemical Analyses

The sections of paraffin-embedded tissues were deparaffinized and antigen-retrieval was achieved by heating the sections in target retrieval solution (Dako Corporation, Denmark)., Slides were treated with 3% H_2_O_2_ to block endogenous peroxidase activity. Immunostaining with murine monoclonal antibodies was performed using the Vector M.O.M. immunodetection kit (Vector Laboratories, Burlingham, Calif.). Staining with rat antibodies was performed using the VECTASTAIN Elite ABC Kit (Vector Laboratories), and staining with rabbit antibodies was performed using the Dako REAL EnVision Detection System, rabbit/mouse kit (Dako Corporation, Denmark). After blocking, slides were incubated with primary antibodies listed in Table 1. Reaction products were identified by immersing the slides in diaminobenzidine tetrachloride (DAB) to yield a brown reaction product. The slides were then counterstained with hematoxylin and mounted. Negative controls included replacement of the primary antibody by irrelevant isotype-matched antibodies. Collagen content was analyzed by picrosirius red and polarized light microscopic imaging. Immunohistochemical or picrosirius red staining was quantified by Photoshop-based image analysis as described [25,26]. The ratio of the positively stained area to the total lesion area (percent-positive area) or the number of positively stained cells per mm^2^ lesion, respectively, was calculated. All quantitative morphometric and immunohistochemical data were collected independently by two experienced operators blinded to the mice genotypes.

**Table 1:** Primary Antibodies for Immunohistochemistry.

Antibodies	Name	Source	Company
α-Actin	human Muscle Actin (HHF35) mAb	Mouse	Dako
cCaspase3	Cleaved Caspase-3 (Asp175) Antibody	Rabbit	Cell Signaling
CD2	Anti-CD2 polyclonal antibody	Rabbit	Bioss
CD56	NCAM1/CD56 polyclonal antibody	Rabbit	Proteintech
CD57	Anti-CD57 rabbit clonal antibody	Rabbit	DB Biotech
F4/80	Anti-mouse F4/80 (CI:A3-1) mAb	Rat	AbD Serotec
Geminin	Geminin Polyclonal Antibody	Rabbit	Proteintech
VCAM1	Anti-VCAM1 antibody (EPR5047)	Rabbit	Abcam

mAb: monoclonal antibody

### Statistical Analyses

Data were analysed with Prism 5.0. Most of the outcome parameters determined in this study (macrophage, T-, NK- and smooth muscle (SMC) cellularity as well as collagen content of atherosclerotic lesions) did not follow a normal distribution as judged by Shapiro-Wilk tests. These parameters are thus presented as box-plots with median, interquartile range, minimum and maximum diagrams and their statistical analyses have been performed with the non-parametric Mann-Whitney *U* tests. Body weights and serum lipid concentrations were found to follow a normal distribution. These data are thus presented as mean (± SD) and were analyzed by t-test of significance. Differences between the mouse genotypes were considered as significant for p-values < 0.05.

## Results

### Serum Lipids and Lipoproteins

**Fig 1:**
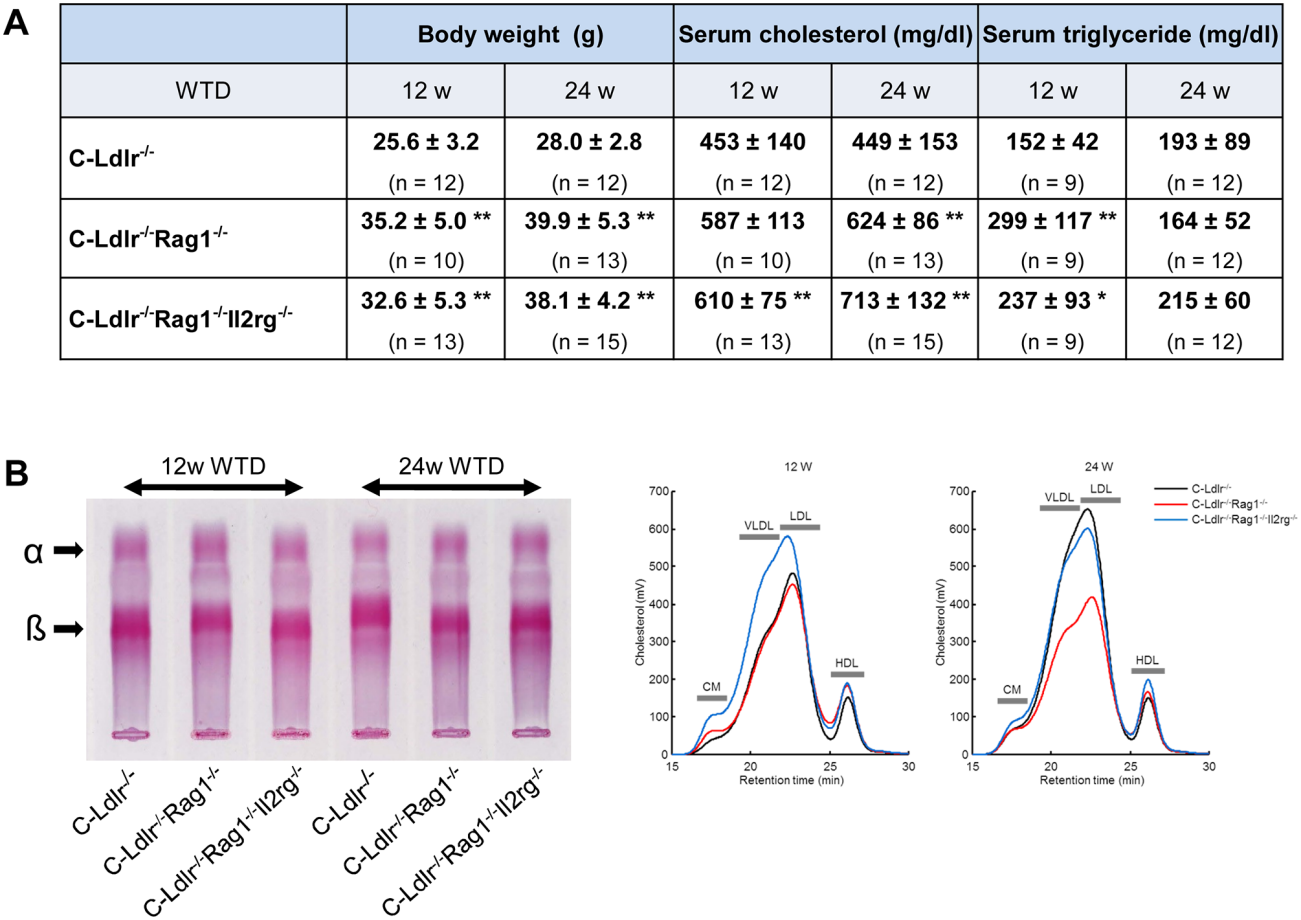
Lipid and Lipoprotein analysis of murine sera. **A,** Group size (n), body weights,serum cholesterol and serum triglyceride concentrations of C-Ldlr-/- controls, C-Ldlr-/- Rag1-/- and C-Ldlr-/- Rag1-/- Il2rg-/-mice after 12 and 24 weeks (w) on the WTD, respectively. Data are presented as means ± standard deviations. *, ** indicate statistically significant differences (*p < 0.05, ** p<0.01). **B,** Agarose gel electrophoresis of serum lipoproteins (left panel). Serum (2 μL) was electrophoresed on a 1% agarose gel and stained for neutral lipids (α- and ß-migrating lipoproteins) with Fat Red 7B. Representative lipoprotein profiles (right panel). Pooled plasma (n = 6) collected from mice fed a WTD for 12 weeks was used and analyzed by FPLC as described in the Materials and Methods.

Starting at eight weeks of age female C-Ldlr^-/-^ Rag1^-/-^ and C-Ldlr^-/-^ Rag1^-/-^ Il2rg^-/-^ knockout mice, as well as female C-Ldlr^-/-^ and B6-Ldlr^-/-^ controls were administered an atherogenic WTD for 12 or 24 weeks, respectively. Fig 1A shows the group sizes, body weights, serum cholesterol and triglyceride levels of all genotypes and of the various dietary groups. Body weights and serum cholesterol levels of both C-Ldlr^-/-^ Rag1^-/-^ and C-Ldlr^-/-^ Rag1^-/-^ Il2rg^-/-^ immunodeficient strains were significantly increased compared to C-Ldlr^-/-^ controls (p<0.01), respectively, except for cholesterol levels of C-Ldlr^-/-^ Rag1^-/-^ double mutants after 12 weeks on the WTD. Moreover, cholesterol levels of C-Ldlr^-/-^ Rag1^-/-^ Il2rg^-/-^ triple mutants were higher than those of C-Ldlr^-/-^ Rag1^-/-^ double mutants after 24 weeks on the WTD (p<0.05). Serum triglyceride levels of both C-Ldlr^-/-^ Rag1^-/-^ and C-Ldlr^-/-^ Rag1^-/-^ Il2rg^-/-^ immunodeficient strains were significantly increased compared to C-Ldlr^-/-^ controls after 12 weeks on the WTD (p<0.01 and p<0.05, respectively). However, there, were no significant differences in triglyceride levels after 24 weeks on the WTD.

To analyze the effect of immunodeficiency on lipoproteins, serum lipoprotein profiles were examined by both agarose gel electrophoresis followed by staining with Fat Red 7B after (Fig 1B, left panel) and FPLC (Fig 1B, right panel). It appears that apoB-containing particles (chylomicrons, VLDLs and LDLs) were increased in triple mutant mice but HDL contents were unchanged compared with other two groups.

### Atherosclerosis Lesion Development

**Fig 2:**
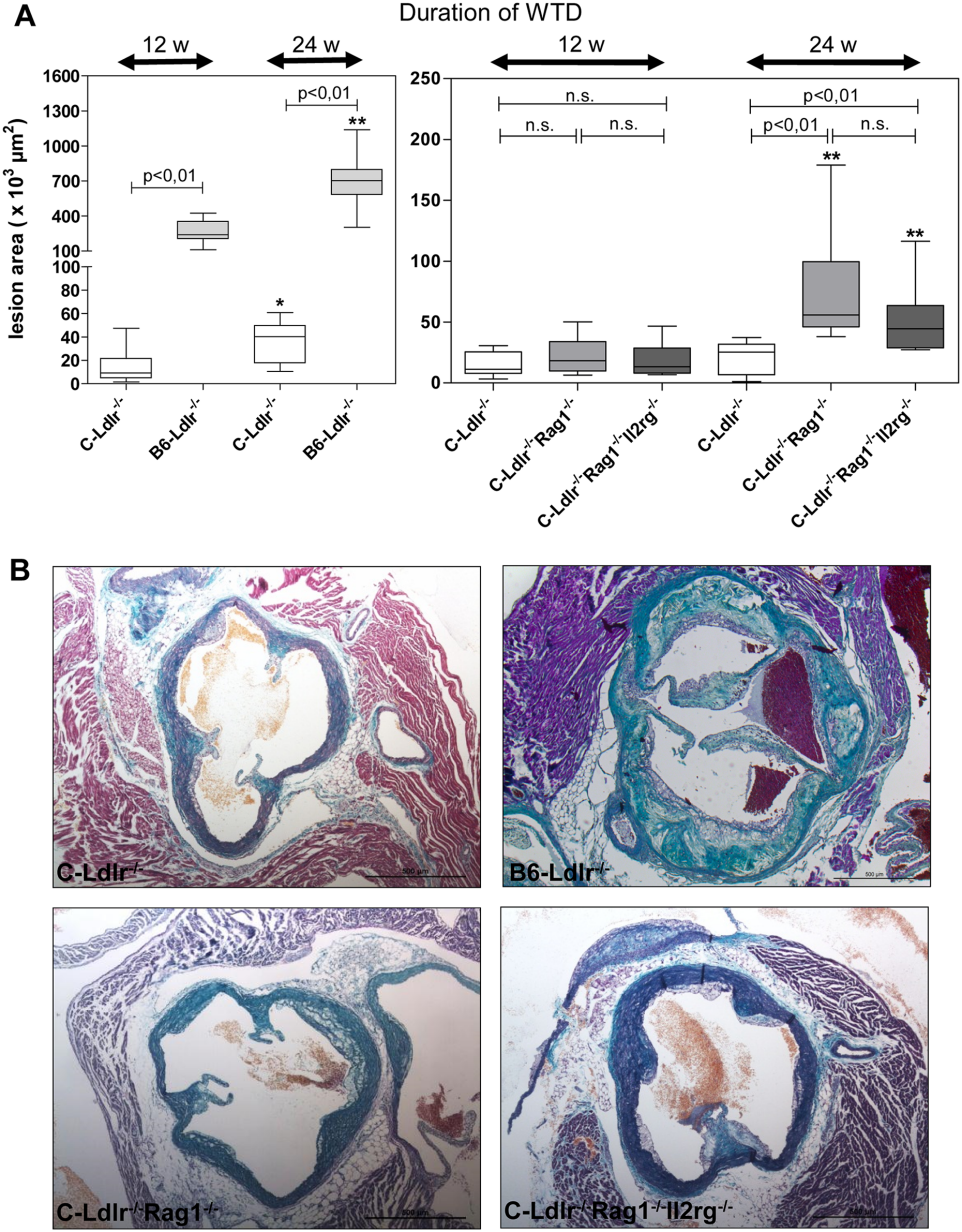
Atherosclerotic lesion development. **A,** Quantification of cross-sections of the aortic sinus area of B6-Ldlr^-/-^ and C-Ldlr^-/-^ mice (left panel), C-Ldlr^-/-^, C-Ldlr^-/-^ Rag1^-/-^ and C-Ldlr^-/-^ Rag1^-/-^ Il2rg^-/-^ mice (right panel) after 12 weeks (12 w), and 24 weeks (24 w) on the WTD, respectively. Lesion plaque areas were presented as boxplots with median, interquartile range, minimum, and maximum. *, ** indicate statistically significant differences (* p < 0.05, ** p<0.01), n. s. = not significant. **B,** Representative specimens of the aortic sinus area of C-Ldlr^-/-^ (upper left panel), B6-Ldlr^-/-^ (upper right panel), C-Ldlr^-/-^ Rag1^-/-^ (lower left panel) and C-Ldlr^-/-^ Rag1^-/-^ Il2rg^-/-^ (lower right panel) mice after 24 weeks on the WTD (trichrome staining).

Mice were sacrificed after 12 or 24 weeks on the WTD. We first evaluated whether atherosclerotic lesion development could be observed in mice on the BALB/c background (Fig 2A, left panel). As expected, quantification of the plaque areas (median/interquartile range) of the aortic sinus revealed significant more lesion development in B6-Ldlr^-/-^ mice (12 weeks: 230343/184909, 24 weeks: 702682/217326 μm^2^) compared to C-Ldlr^-/-^ mice (12 weeks: 9195/16769, 24 weeks: 40206/32221 μm^2^). Of note, however, there was sufficient lesion development in C-Ldlr^-/-^ mice after both 12 and 24 weeks on the WTD.

Next, we compared the two immunodeficient strains with the C-Ldlr^-/-^ controls. As demonstrated in the right panel of Fig 2A and in the lower panels of Fig 2B, substantial lesions developed in the aortic sinus area of all animals and the lesions increased in size with time. There were no quantitative differences between the cohorts after 12 weeks on the WTD. Quantification of the plaque areas (median/interquartile range) of the aortic sinus after 24 weeks on the WTD, however, revealed that C-Ldlr^-/-^ Rag1^-/-^ (55916/53488 μm^2^) and C-Ldlr^-/-^ Rag1^-/-^ Il2rg^-/-^ (44522/34736 μm^2^) immunodeficent mice developed significantly more atherosclerosis compared to C-Ldlr^-/-^ controls (25285/24975 μm^2^, p<0,01, Fig 2A, right panel). Representative photomicrographs of the atherosclerotic lesions observed are shown in Fig 2B.

### Phenotype Analysis of Atherosclerotic Lesions

**Fig 3:**
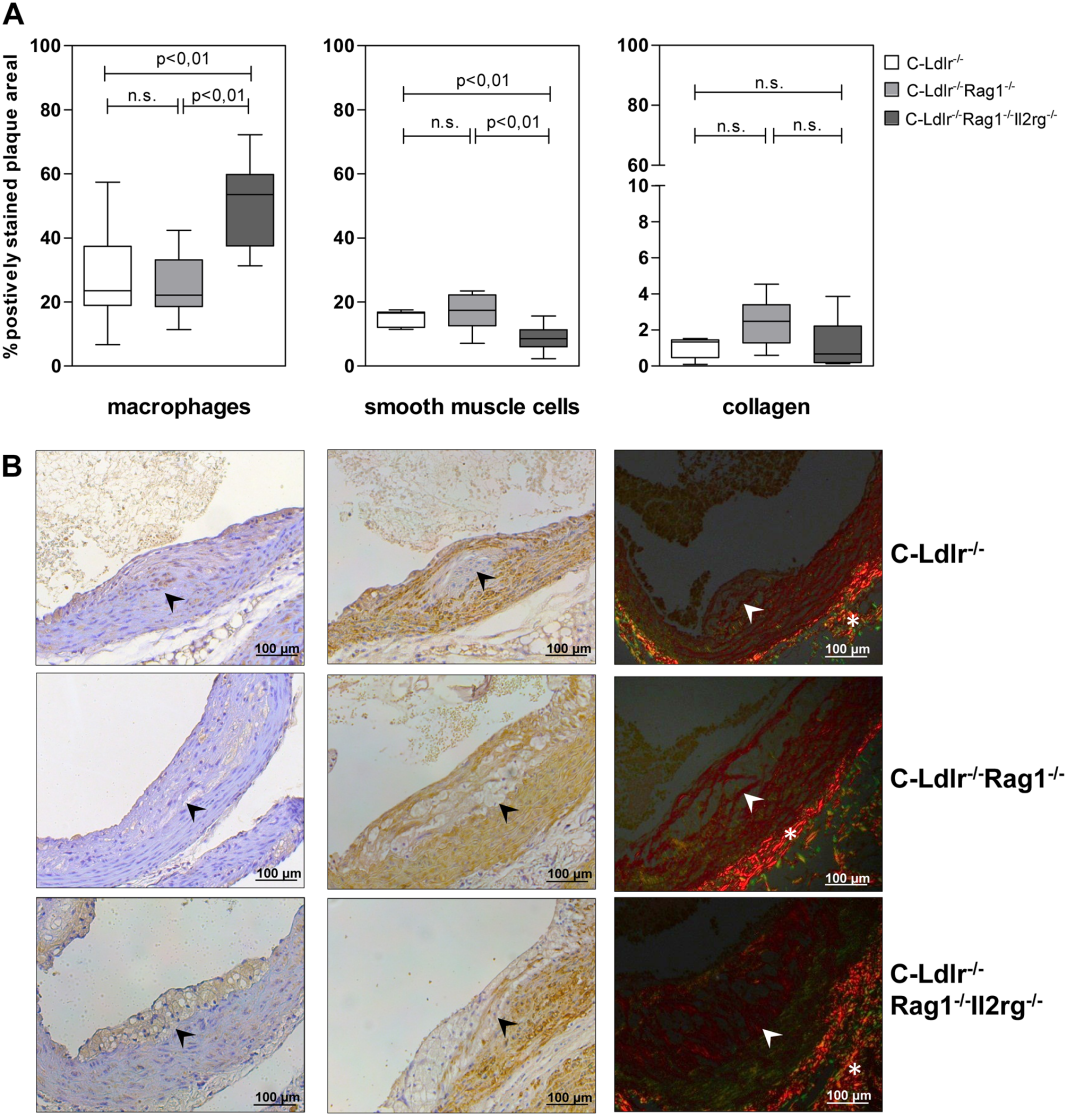
Phenotypic analysis of atherosclerotic lesions. **A,** Analysis of atherosclerotic lesions after 24 weeks (24 w) on the WTD. Atherosclerotic lesions of the aortic sinus of C-Ldlr^-/-^ controls, C-Ldlr^-/-^ Rag1^-/-^ and C-Ldlr^-/-^ Rag1^-/-^ Il2rg^-/-^ mice were quantified for macrophages (left panel), SMCs (middle panel), and collagen (right panel). Percent-positive area for macrophages, SMCs, and collagen was quantified by Photoshop-based image analysis. Data are presented as boxplots with median, interquartile range, minimum, and maximum. **B,** Representative examples of atherosclerotic lesion composition after 24 weeks on the WTD. Atherosclerotic lesions of the aortic sinus of C-Ldlr^-/-^ controls, C-Ldlr^-/-^ Rag1^-/-^ and C-Ldlr^-/-^ Rag1^-/-^ Il2rg^-/-^ mice were stained with rat anti-mouse F4/80 mAb (left panels) for quantification of macrophages, mouse anti-smooth muscle α-actin (middle panels) for quantification of SMCs, and picrosirius red with subsequent polarization (right panels) for quantification of collagen. The vessel lumen is to the upper left corner. The demarcation between intima and media is indicated by arrowheads. Note that the adventitial tissue (*) also polarizes after picrosirius red staining (internal positive control).

**Fig 4:**
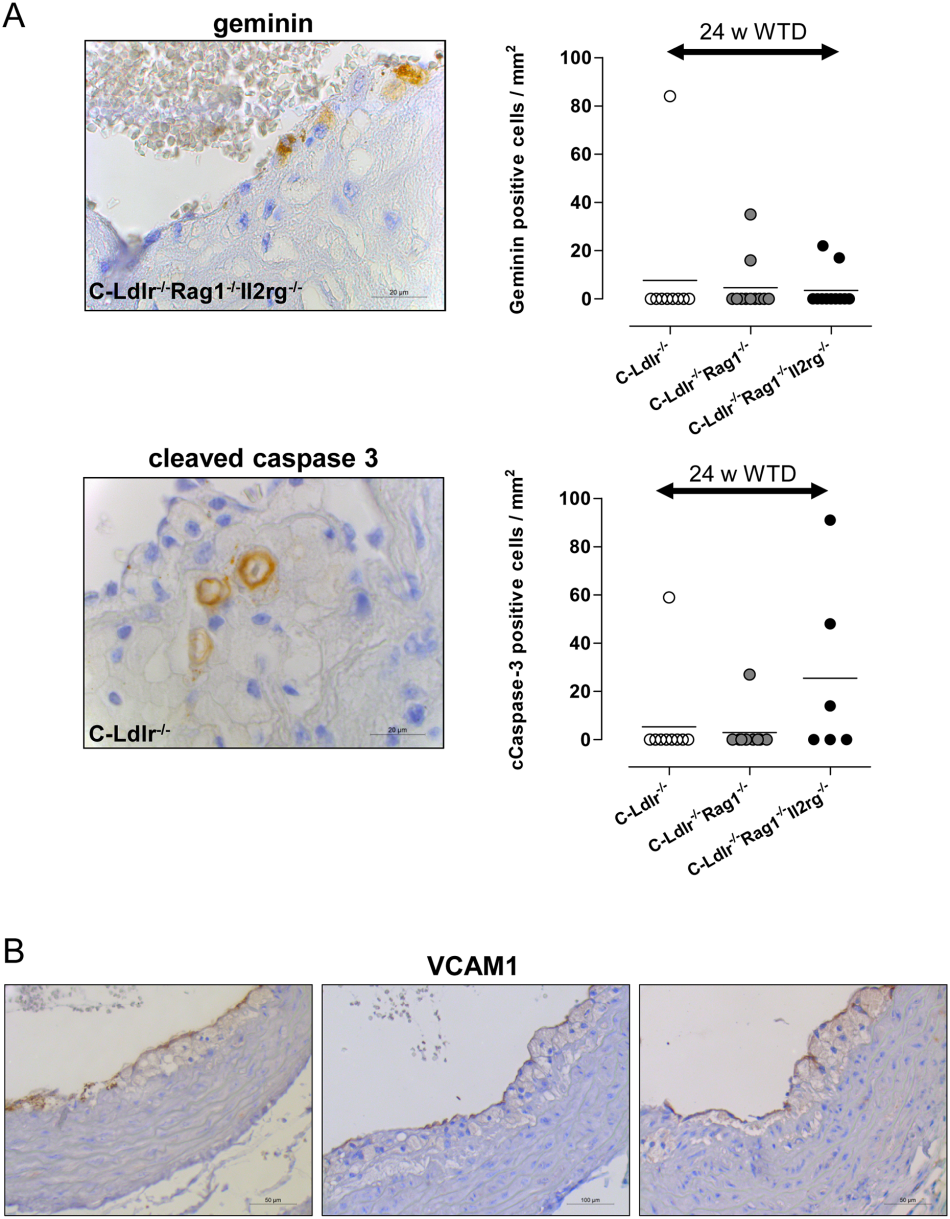
Proliferation, apoptosis and VCAM-1 expression in atherosclerotic lesions. **A**, Proliferation and apoptosis. Left panels, Representative immunohistochemical staining with rabbit polyclonal antibodies against geminin (upper panel) or cleaved caspase 3 (lower panel) in atherosclerotic lesions of the aortic sinus. The aortic lumen is to the upper left corner. Right panels, The number of positively stained cells per atherosclerotic lesion area (mm^2^) was determined for both geminin (upper panel) and cleaved caspase 3 (lower panel). Note high interindividual variations without any significant differences between the different mouse strains. **B**, VCAM-1 expression. Representative immunohistochemical staining with rabbit monoclonal antibody against VCAM-1 in atherosclerotic lesions of the aortic sinus. Note VCAM-1 expression in endothelial cells with no significant differences between the three animal groups. The aortic lumen is to the upper left corner.

Since immunodeficiency obviously influenced atherosclerosis lesion progression in BALB/c mice, we next compared lesion composition (percent of macrophages, SMCs, number of T- and NK cells, and collagen per aortic sinus area) in the immunodeficient mutants and C-Ldlr^-/-^ controls. However, due to extreme outliers and high interindividual variations at this early time point, we abstained from further phenotype analysis of atherosclerotic lesions at 12 weeks and confined to phenotype analysis after 24 weeks on the WTD (Figs 3 and 4). The macrophage and SMC cellularity as well as collagen content were not significantly different between C-Ldlr^-/-^ Rag1^-/-^ double mutants and C-Ldlr^-/-^ controls (Fig 3A). Interestingly, increased atherosclerotic lesion development in C-Ldlr^-/-^ Rag1^-/-^ Il2rg^-/-^ triple mutants was associated with significantly more macrophages (53,6/22,3%, Fig 3A, left panel), significantly less SMCs (8,5/5,3%, p<0.01, Fig 3A, middle panel) without significantly differences in collagen (1,6/1,9%, Fig 3A, right panel) as compared to both C-Ldlr^-/-^ wild type (macrophages 23,6/18,4%, p<0.01; SMCs 16,5/4,7%, p<0.01; collagen 1,3/1,0%) and C-Ldlr^-/-^ Rag1^-/-^ double mutants (macrophages 22,1/14,6%, p<0.01; SMCs 17,4/9,6%, p<0.01; collagen 2,5/2,1%) suggesting a plaque destabilizing effect of immunodeficiency. These differences, however, were not attributable to different proliferation and /or apoptosis as determined by immunohistochemistry (Fig 4A). Likewise, VCAM-1, a member of the adhesion molecule superfamily, was not expressed differentially in the three animal groups (Fig 4B).

**Fig 5:**
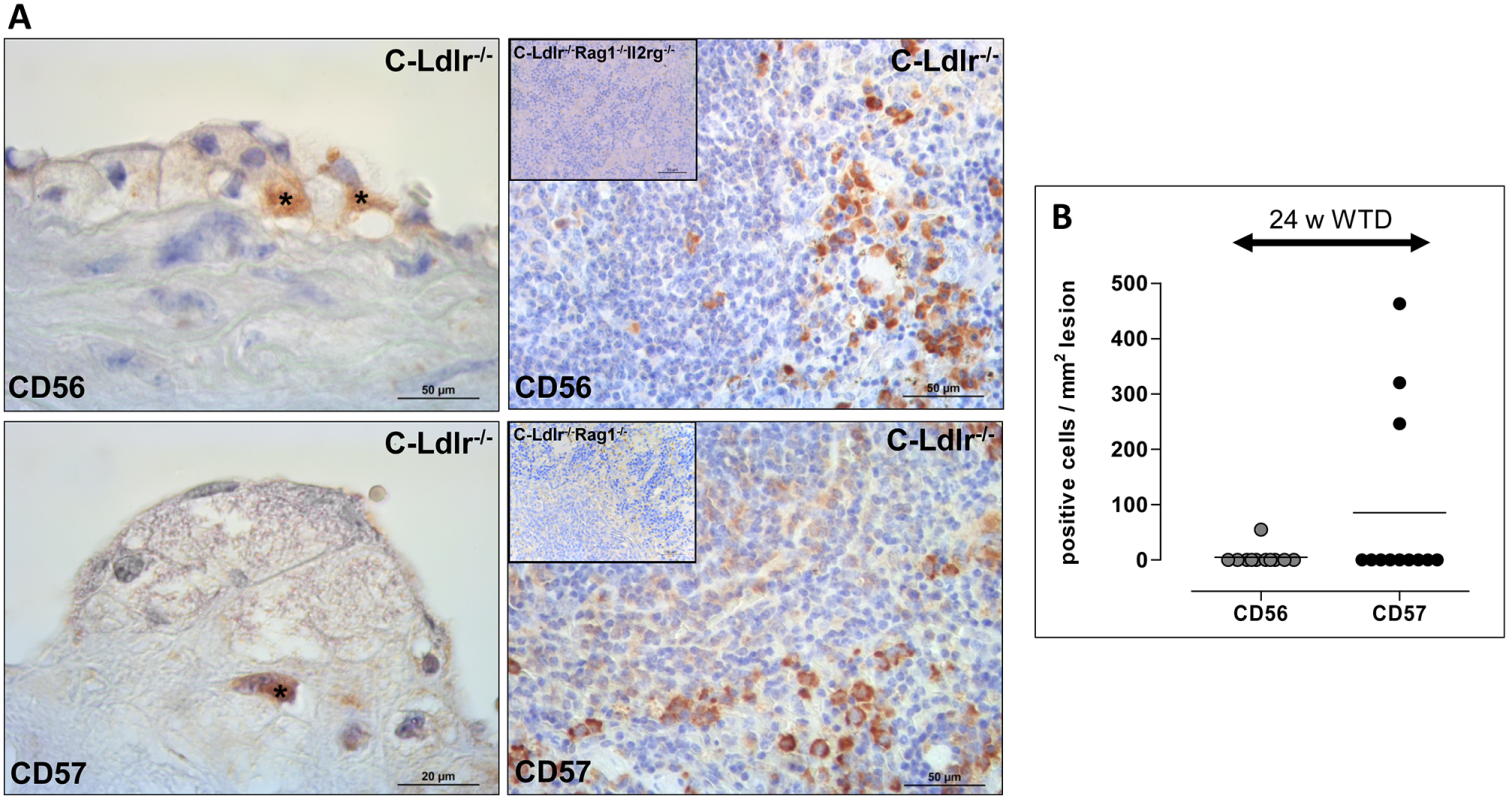
NK and NKT cells in atherosclerotic lesions and spleens of C-Ldlr^-/-^ control mice. Representative immunohistochemical staining with a rabbit polyclonal antibody against CD56 (Proteintech, Manchester, UK) (upper panels) or CD57 (Acris Antibodies, Hiddenhausen, Germany) (lower panels) of an atherosclerotic lesion located in the aortic sinus (left) and the spleen as a positive control (right) **(A)**. Note that Nk cells were detectable neither in double nor in triple mutants (inserts). The number of positively stained cells (asterisks) per atherosclerotic lesion area (mm^2^) was determined **(B)**. The aortic lumen is to the upper left corner.

To evaluate the potential role of T and NK cells, the lesional cellularity of CD2+ (surface antigen of the T lymphocyte lineage, not shown), CD56+ (expressed on NK cells and a subset of T cells) and CD57+ (expressed on a subset of NK cells and a subset of T lymphocytes) cells was investigated (Fig 5). However, on average, there were only insignificant numbers or no cells except for three mice with higher numbers of CD57+ cells (Fig 5B). Basically, Nk cells were not detectable in both double and triple mutants (Fig 5A, inserts).

## Discussion

In the present study, a combined B and T cell immunodeficiency with or without a complete lack of natural killer cells in Th2-prone C-Ldlr knockout mice significantly accelerates atherosclerotic lesion progression after 24 weeks on a WTD. Furthermore, a combined B and T cell immunodeficiency with a complete lack of natural killer cells has the potential to change atherosclerotic cellularity with more macrophages and fewer SMCs. Strikingly, these effects were accompanied by significantly higher body weights as well as serum cholesterol and triglyceride levels in the immunodeficient mice of both strains. Increased serum lipids were found to be caused by elevated apoB-containing lipoproteins such as VLDLs and LDLs as shown in FPLC results. It is currently unknown; however, how immunodeficiency affects lipoprotein metabolism such as increased production of VLDL particles or delayed catabolism of these particles in the liver. In spite of this, immunodeficiency did not influence systemic cytokine levels.

At a first glance, these results appear to be contradictory to several previous studies investigating the impact of immunodeficiency on atherogenesis [2–4]. An alternate point of view, however, modifies this first impression and points to some new interesting issues concerning immunodeficiency and atherogenesis. First of all, the significantly higher serum cholesterol and triglyceride levels in the immunodeficient mice of both strains are striking. These differences may be attributed, at least in part, to the BALB/c background. Besides C57BL/6 mice, BALB/c mice are widely used throughout biomedical research with a high susceptibility to atherogenesis following a high fat diet. Thus, depletion of NKT cells resulted in an obese phenotype with insulin resistance, glucose intolerance, body weight gain and lipid accumulation [27]. Another study showed increased triglyceride content, liver injury and inflammation upon Type I NKT depletion [28]. It has previously been demonstrated that thymic NKT cells of BALB/c mice express higher levels of IL-4, which has been linked to increased protection from metabolic dysregulation compared to C57BL/6 mice [29]. Given the different Th1/Th2 balance of C57BL/6 and BALB/c mice, the loss of IL-4 may lead to a more severe phenotype in the latter. Preliminary results of cytokine analyses in the murine sera, however, suggest that the proposed cytokine effects are mediated locally rather than systemically, e. g. by adipose-derived NKT cells [30].

Quite similar observations have been also made on the C57BL/6 background. Thus, adipose tissue invariant NKT cells protected against diet-induced obesity and metabolic disorder through regulatory cytokine production [30]. In another previous study investigating the role of NKT cells in an adoptive transfer model of atherosclerosis using immunodeficient, atherosclerosis-susceptible B6-Rag1^-/-^ Ldlr^-/-^ mice as recipients, significantly higher plasma total cholesterol and triglyceride levels were observed in the recipients of B6-CD1d^-/-^ splenocytes compared with the recipients of B6 splenocytes at the terminal 12-week time point [10]. Furthermore, it cannot be excluded that a lack of NK cells directly raises serum lipoproteins as the latter have been shown to be internalized by NK cells through specific LDL-receptor mediated uptake [31–33].

The significantly higher body weights and serum cholesterol levels in the immunodeficient mice of both strains may underlie the accelerated atherosclerotic lesion progression. Another impact may be attributed to the B cell immunodeficiency, as innate B-1 cells have been demonstrated to play a protective role in atherogenesis both in Apoe- [5,34] and Ldlr-deficient [6] mice through the secretion of naturally occurring antibodies.

Of course, T cells also play a role in atherogenesis. An earlier study using a class I MHC deficient C57BL/6J strain has already shown that a lack of suppressor T cells was associated with an increase in lesion formation [35]. Since then, a plethora of studies provided dual roles for T lymphocytes in both suppressing and promoting atherosclerosis depending on different Th-cell polarization (Th1, Th2, Th17, and Treg), predictive for the specific type of immune response [11]. A matter of particular interest is the recent observation that depletion of FOXP3+ regulatory T cells promotes hypercholesterolemia and atherosclerosis in Ldlr^-/-^ deficient mice [36]. As our animal models orchestrate several of these possible mechanisms simultaneously, it is difficult to address causality for the unexpected altered lipoprotein levels. This should be subject to more sophisticated adoptive transfer animal models.

As we have demonstrated, the modulating effect of the C-Ldlr^-/-^ Rag1^-/-^ Il2rg^-/-^ triple mutants on atherosclerotic cellularity with more macrophages and fewer SMCs was neither attributable to different proliferation and/or apoptosis nor to different adhesion capacities of the endothelium mediated by different VCAM-1 expression. Rather, this phenomenon argues for a more sophisticated view of the impact of NK cells on atherogenesis. Schulz et al. showed in their recent publication that NK cell cytotoxicity against autologous macrophages remains elevated if cultured with IL-10 [37]. The authors suggest that NK cells can delete macrophages and potentially other immature APC under inflammatory conditions that could be a possible mechanism of macrophage increase in triple KO atherosclerotic lesions. Compared to control and double KO mice (24 weeks on WTD), triple KO mice obviously developed atherosclerotic lesions with morphological features of fatty streak-like lesions. These types of early lesions do not develop fibrous caps and always have a very high presentation of macrophages. If so, lack of NK cells in triple KO mice leads to slowing down atherogenesis, development of fatty streak-like lesions, and as a result—increase of macrophage content in this experimental group consistent with the observation that lesion area of double and triple mutants is not vastly different (Fig 2A) as against macrophage cellularity (Fig 3A). Accordingly, NK cells have been shown to promote atherosclerotic lesion development in Ldlr^-/-^ and Apoe^-/-^ mice, respectively [38,39]. Whatever the precise mechanisms are, it seems unlikely that this modulating effect can be attributed to NK cells *within* the atherosclerotic lesions as we found only very limited numbers of NK and NKT cells in the Ldlr^-/-^ control mice using two different antibodies (CD56, CD57). Rather, systemic effects such as the impact of the total NK and NKT cell pool on lipoprotein metabolism (see above) should be considered.

It is now widely accepted that immune cells play an important role in atherogenesis. Collectively, the present study reveals a previously unappreciated complexity with regard to the impact of lymphocytes on lipoprotein metabolism on the one hand and the role of certain lymphocyte subsets in plaque composition on the other hand. This complexity may be attributed, at least in part, to the Th2-prone BALB/c background. BALB/c mice homozygous for targeted immunological mutations like Rag1-knockout, Rag2-knockout and the Il2rg-knockout are suitable recipients for human hematopoetic stem cells and therefore the study of atherosclerosis in humanized mice.

## Supporting Information

S1 Table Summary statistics for body weights, serum cholesterol and triglyceride levels of all mouse groups. (XLSX)

S2 Table Quantification of the plaque areas of the aortic sinus of all mouse groups. (XLSX)

S3 Table Phenotypic analysis of atherosclerotic lesions in C-Ldlr^-/-^, C-Ldlr^-/-^ Rag1^-/-^ and C-Ldlr^-/-^ Rag1^-/-^ Il2rg^-/-^ mice after 24 weeks on the western type diet. (XLSX)

S4 Table Quantification of cell proliferation and apoptosis in atherosclerotic lesions of C-Ldlr^-/-^, C-Ldlr^-/-^ Rag1^-/-^ and C-Ldlr^-/-^ Rag1^-/-^ Il2rg^-/-^ mice as determined by immunohistochemistry. (XLSX)

S5 Table Quantification of NK cell distribution in atherosclerotic lesions of C-Ldlr^-/-^ mice as determined by immunohistochemistry. (XLSX)

## References

[1] Libby P, Lichtman AH, Hansson GK (2013) Immune effector mechanisms implicated in atherosclerosis: from mice to humans. Immunity 38: 1092–1104. 10.1016/j.immuni.2013.06.009 23809160 PMC3764500

[2] Daugherty A, Pure E, Delfel-Butteiger D, Chen S, Leferovich J, Roselaar SE, et al (1997) The effects of total lymphocyte deficiency on the extent of atherosclerosis in apolipoprotein E-/- mice. J Clin Invest 100: 1575–1580. 9294126 10.1172/JCI119681 PMC508339

[3] Dansky HM, Charlton SA, Harper MM, Smith JD (1997) T and B lymphocytes play a minor role in atherosclerotic plaque formation in the apolipoprotein E-deficient mouse. Proc Natl Acad Sci U S A 94: 4642–4646. 9114044 10.1073/pnas.94.9.4642 PMC20777

[4] Song L, Leung C, Schindler C (2001) Lymphocytes are important in early atherosclerosis. J Clin Invest 108: 251–259. 11457878 10.1172/JCI11380 PMC203020

[5] Caligiuri G, Nicoletti A, Poirier B, Hansson GK (2002) Protective immunity against atherosclerosis carried by B cells of hypercholesterolemic mice. J Clin Invest 109: 745–753. 11901183 10.1172/JCI07272 PMC150903

[6] Major AS, Fazio S, Linton MF (2002) B-lymphocyte deficiency increases atherosclerosis in LDL receptor-null mice. Arterioscler Thromb Vasc Biol 22: 1892–1898. 12426221 10.1161/01.atv.0000039169.47943.ee

[7] Getz GS, Vanderlaan PA, Reardon CA (2011) Natural killer T cells in lipoprotein metabolism and atherosclerosis. Thromb Haemost 106: 814–819. 10.1160/TH11-05-0336 21946866 PMC3696188

[8] Nakai Y, Iwabuchi K, Fujii S, Ishimori N, Dashtsoodol N, Watano K, et al (2004) Natural killer T cells accelerate atherogenesis in mice. Blood 104: 2051–2059. 15113755 10.1182/blood-2003-10-3485

[9] Aslanian AM, Chapman HA, Charo IF (2005) Transient role for CD1d-restricted natural killer T cells in the formation of atherosclerotic lesions. Arterioscler Thromb Vasc Biol 25: 628–632. 15591216 10.1161/01.ATV.0000153046.59370.13

[10] VanderLaan PA, Reardon CA, Sagiv Y, Blachowicz L, Lukens J, Nissenbaum M, et al (2007) Characterization of the natural killer T-cell response in an adoptive transfer model of atherosclerosis. Am J Pathol 170: 1100–1107. 17322392 10.2353/ajpath.2007.060188 PMC1864866

[11] Ait-Oufella H, Sage AP, Mallat Z, Tedgui A (2014) Adaptive (T and B cells) immunity and control by dendritic cells in atherosclerosis. Circ Res 114: 1640–1660. 10.1161/CIRCRESAHA.114.302761 24812352

[12] Zhou X, Paulsson G, Stemme S, Hansson GK (1998) Hypercholesterolemia is associated with a T helper (Th) 1/Th2 switch of the autoimmune response in atherosclerotic apo E-knockout mice. J Clin Invest 101: 1717–1725. 9541503 10.1172/JCI1216 PMC508754

[13] Schulte S, Sukhova GK, Libby P (2008) Genetically programmed biases in Th1 and Th2 immune responses modulate atherogenesis. Am J Pathol 172: 1500–1508. 10.2353/ajpath.2008.070776 18467709 PMC2408411

[14] Roselaar SE, Daugherty A (1998) Apolipoprotein E-deficient mice have impaired innate immune responses to Listeria monocytogenes in vivo. J Lipid Res 39: 1740–1743. 9741685

[15] Ishibashi S, Brown MS, Goldstein JL, Gerard RD, Hammer RE, Herz J (1993) Hypercholesterolemia in low density lipoprotein receptor knockout mice and its reversal by adenovirus-mediated gene delivery. J Clin Invest 92: 883–893. 8349823 10.1172/JCI116663 PMC294927

[16] Piedrahita JA, Zhang SH, Hagaman JR, Oliver PM, Maeda N (1992) Generation of mice carrying a mutant apolipoprotein E gene inactivated by gene targeting in embryonic stem cells. Proc Natl Acad Sci U S A 89: 4471–4475. 1584779 10.1073/pnas.89.10.4471 PMC49104

[17] Zhang SH, Reddick RL, Piedrahita JA, Maeda N (1992) Spontaneous hypercholesterolemia and arterial lesions in mice lacking apolipoprotein E. Science 258: 468–471. 1411543 10.1126/science.1411543

[18] Spencer MW, Mühlfeld AS, Segerer S, Hudkins KL, Kirk E, LeBoeuf RC, et al (2004) Hyperglycemia and hyperlipidemia act synergistically to induce renal disease in LDL receptor-deficient BALB mice. Am J Nephrol 24: 20–31. 14671436 10.1159/000075362

[19] Mombaerts P, Iacomini J, Johnson RS, Herrup K, Tonegawa S, Papaioannou VE (1992) RAG-1-deficient mice have no mature B and T lymphocytes. Cell 68: 869–877. 1547488 10.1016/0092-8674(92)90030-g

[20] Cao X, Shores EW, Hu-Li J, Anver MR, Kelsall BL, Russell SM, et al (1995) Defective lymphoid development in mice lacking expression of the common cytokine receptor gamma chain. Immunity 2: 223–238. 7697543 10.1016/1074-7613(95)90047-0

[21] Petkov PM, Ding Y, Cassell MA, Zhang W, Wagner G, Sargent EE, et al (2004) An efficient SNP system for mouse genome scanning and elucidating strain relationships. Genome Res 14: 1806–1811. 15342563 10.1101/gr.2825804 PMC515327

[22] Koike T, Kitajima S, Yu Y, Li Y, Nishijima K, Liu E, et al (2009) Expression of human apoAII in transgenic rabbits leads to dyslipidemia: a new model for combined hyperlipidemia. Arterioscler Thromb Vasc Biol 29: 2047–2053. 10.1161/ATVBAHA.109.190264 19778946

[23] Fan J, Unoki H, Kojima N, Sun H, Shimoyamada H, Deng H, et al (2001) Overexpression of lipoprotein lipase in transgenic rabbits inhibits diet-induced hypercholesterolemia and atherosclerosis. J Biol Chem 276: 40071–40079. 11477088 10.1074/jbc.M105456200

[24] Paigen B, Morrow A, Holmes PA, Mitchell D, Williams RA (1987) Quantitative assessment of atherosclerotic lesions in mice. Atherosclerosis 68: 231–240. 3426656 10.1016/0021-9150(87)90202-4

[25] Lehr HA, Mankoff DA, Corwin D, Santeusanio G, Gown AM (1997) Application of photoshop-based image analysis to quantification of hormone receptor expression in breast cancer. J Histochem Cytochem 45: 1559–1565. 9358857 10.1177/002215549704501112

[26] Torzewski M, Ochsenhirt V, Kleschyov AL, Oelze M, Daiber A, Li H, et al (2007) Deficiency of glutathione peroxidase-1 accelerates the progression of atherosclerosis in apolipoprotein E-deficient mice. Arterioscler Thromb Vasc Biol 27: 850–857. 17255533 10.1161/01.ATV.0000258809.47285.07

[27] Martin-Murphy BV, You Q, Wang H, De La Houssaye BA, Reilly TP, Friedman JE, et al (2014) Mice lacking natural killer T cells are more susceptible to metabolic alterations following high fat diet feeding. PLoS One 9: e80949 10.1371/journal.pone.0080949 24465369 PMC3896335

[28] Miyagi T, Takehara T, Uemura A, Nishio K, Shimizu S, Kodama T, et al (2010) Absence of invariant natural killer T cells deteriorates liver inflammation and fibrosis in mice fed high-fat diet. J Gastroenterol 45: 1247–1254. 10.1007/s00535-010-0272-y 20596733

[29] Lai D, Zhu J, Wang T, Hu-Li J, Terabe M, Berzofsky JA, et al (2011) KLF13 sustains thymic memory-like CD8(+) T cells in BALB/c mice by regulating IL-4-generating invariant natural killer T cells. J Exp Med 208: 1093–1103. 10.1084/jem.20101527 21482696 PMC3092346

[30] Lynch L, Nowak M, Varghese B, Clark J, Hogan AE, Toxavidis V, et al (2012) Adipose tissue invariant NKT cells protect against diet-induced obesity and metabolic disorder through regulatory cytokine production. Immunity 37: 574–587. 10.1016/j.immuni.2012.06.016 22981538 PMC4991771

[31] De Sanctis JB, Blanca I, Bianco NE (1995) Expression of different lipoprotein receptors in natural killer cells and their effect on natural killer proliferative and cytotoxic activity. Immunology 86: 399–407. 8550077 PMC1383943

[32] Juompan L, Fournie GJ, Benoist H (1994) LDL and acetyl-LDL inhibit the NK activity and are taken up by CD56+ lymphocytes. Biochim Biophys Acta 1224: 1–10. 7524682 10.1016/0167-4889(94)90106-6

[33] Maczek C, Recheis H, Böck G, Stulnig T, Jürgens G, Wick G (1996) Comparison of low density lipoprotein uptake by different human lymphocyte subsets: a new method using double-fluorescence staining. J Lipid Res 37: 1363–1371. 8808771

[34] Chen A, Geng Y, Ke H, Constant L, Yan Z, Pan Y, et al (2014) Cutting edge: Dexamethasone potentiates the responses of both regulatory T cells and B-1 cells to antigen immunization in the ApoE(-/-) mouse model of atherosclerosis. J Immunol 193: 35–39. 10.4049/jimmunol.1302469 24899497 PMC4153946

[35] Fyfe AI, Qiao JH, Lusis AJ (1994) Immune-deficient mice develop typical atherosclerotic fatty streaks when fed an atherogenic diet. J Clin Invest 94: 2516–2520. 7989611 10.1172/JCI117622 PMC330086

[36] Klingenberg R, Gerdes N, Badeau RM, Gisterå A, Strodthoff D, Ketelhuth DF, et al (2013) Depletion of FOXP3+ regulatory T cells promotes hypercholesterolemia and atherosclerosis. J Clin Invest 123: 1323–1334. 10.1172/JCI63891 23426179 PMC3582120

[37] Schulz U, Kreutz M, Multhoff G, Stoelcker B, Kohler M, Andreesen R, et al (2010) Interleukin-10 promotes NK cell killing of autologous macrophages by stimulating expression of NKG2D ligands. Scand J Immunol 72: 319–331. 10.1111/j.1365-3083.2010.02435.x 20883317

[38] Whitman SC, Rateri DL, Szilvassy SJ, Yokoyama W, Daugherty A (2004) Depletion of natural killer cell function decreases atherosclerosis in low-density lipoprotein receptor null mice. Arterioscler Thromb Vasc Biol 24: 1049–1054. 14988092 10.1161/01.ATV.0000124923.95545.2c

[39] Selathurai A, Deswaerte V, Kanellakis P, Tipping P, Toh BH, Bobik A, et al (2014) Natural killer (NK) cells augment atherosclerosis by cytotoxic-dependent mechanisms. Cardiovasc Res 102: 128–137. 10.1093/cvr/cvu016 24469537

